# Age and Comorbidity Burden of Patients Critically Ill with COVID-19 Affect Both Access to and Outcome of Ventilation Therapy in Intensive Care Units

**DOI:** 10.3390/jcm12072469

**Published:** 2023-03-24

**Authors:** Marie Louise de Hesselle, Stefan Borgmann, Siegbert Rieg, Jörg Janne Vehreschild, Sebastian Rasch, Carolin E. M. Koll, Martin Hower, Melanie Stecher, Daniel Ebert, Frank Hanses, Julia Schumann, on behalf of the LEOSS Study Group

**Affiliations:** 1 University Clinic and Outpatient Clinic for Anesthesiology and Operative Intensive Care, University Medicine Halle (Saale), 06112 Halle (Saale), Germany; 2 Department of Infectious Diseases and Infection Control, Ingolstadt Hospital, 85049 Ingolstadt, Germany; 3 Department of Medicine II, University of Freiburg, 79106 Freiburg, Germany; 4 Department II of Internal Medicine, Hematology/Oncology, Goethe University Frankfurt, 60323 Frankfurt, Germany; 5 Department I of Internal Medicine, Center for Integrated Oncology Aachen Bonn Cologne Duesseldorf, Faculty of Medicine and University Hospital Cologne, University of Cologne, 50931 Cologne, Germany; 6 German Center for Infection Research (DZIF), Partner Site Bonn-Cologne, 50937 Cologne, Germany; 7 Department of Internal Medicine II, University Hospital Rechts der Isar, School of Medicine, Technical University of Munich, 81675 Munich, Germany; 8 Department of Pneumology, Infectious Diseases, Internal Medicine and Intensive Care, Klinikum Dortmund GmbH, 44137 Dortmund, Germany; 9 Emergency Department and Department for Infection Control and Infectious Diseases, University Hospital Regensburg, 93053 Regensburg, Germany

**Keywords:** COVID-19, SARS-CoV-2, age, comorbidities, intensive care medicine, ventilation, ECMO, mortality

## Abstract

During the COVID-19 pandemic, large numbers of elderly, multimorbid people required treatment in intensive care units. This study investigated how the inherent patient factors age and comorbidity burden affected the treatment strategy and the outcome achieved. Retrospective analysis of data from intensive care patients enrolled in the Lean European Open Survey on SARS-CoV2-Infected Patients (LEOSS) cohort found that a patient’s age and comorbidity burden in fact influenced their mortality rate and the use of ventilation therapy. Evidence showed that advanced age and multimorbidity were associated with the restrictive use of invasive ventilation therapies, particularly ECMO. Geriatric patients with a high comorbidity burden were clustered in the sub-cohort of non-ventilated ICU patients characterized by a high mortality rate. The risk of death generally increased with older age and accumulating comorbidity burden. Here, the more aggressive an applied procedure, the younger the age in which a majority of patients died. Clearly, geriatric, multimorbid COVID-19 patients benefit less from invasive ventilation therapies. This implies the need for a holistic approach to therapy decisions, taking into account the patient’s wishes.

## 1. Introduction

Coronavirus disease 2019 (COVID-19), an infectious disease triggered by severe acute respiratory syndrome coronavirus 2 (SARS-CoV-2), has caused a pandemic. The disorder is characterized by a wide spectrum of clinical manifestations. This heterogeneity in clinical presentation points to host factors as a key to disease severity and progression [1]. Indeed, the elderly adult population and those with comorbidities are disproportionately affected by the COVID-19 pandemic in terms of hospitalizations and mortality [1,2,3,4,5,6]. There is an ongoing debate that the poor outcomes among senior adults may be the consequence of a high prevalence of comorbidities, a weak immune system, and a greater degree of frailty in this population [4,5,7,8]. An in-depth review of published data indicates that biological age, rather than chronological age, may play a role in COVID-19 prognosis [7,9,10,11]. The constriction of physiological reserves combined with an impaired ability to properly respond to acute challenges may translate into an increased susceptibility to stressors, such as a viral infection [5,6,12]. Frailty is not a mandatory component of the aging process. Rather, numerous adults attain a high age without being frail. The frail elderly population represents a specific patient group, which, compared to the general population, is characterized by a compromised immune system, a diminished diversity of the gut microbiota, and a persistent state of inflammation [4,5,11]. Accumulating evidence in the literature suggests that those factors collectively contribute to the severity of the COVID-19 disease and the high mortality rate [4,5,11].

The management of critically ill COVID-19 patients is another influencing factor that is still understudied. The COVID-19 pandemic led to a massive influx of patients into hospitals and especially intensive care units (ICUs). Due to limited ICU capacity, criteria for ICU admission and use of mechanical ventilation or extracorporeal membrane oxygenation (ECMO) were frequently tightened [2,5]. This may have had a particular impact on elderly and comorbid patients. Reports indicate that medical staff awareness of a patient’s advanced age and frailty may result in a curtailment of intensive care measures [2,6,10]. By implication, such special handling of a certain group of patients will affect the treatment outcome.

The disproportionate need for intensive care in frail older adults following SARS-CoV-2 infection contrasts with the limited number of studies that have examined the intensive care management of these patients in detail. The present study aimed to retrospectively highlight the potential influence of patient-specific determinants, i.e., age and comorbidity burden. The primary objective was to determine whether the decision for a ventilation regimen in the ICU was indeed co-determined by these intrinsic patient factors. The secondary objective was to assess whether and to what extent age and comorbidity burden were related to treatment outcome, with a separate assessment for ventilation regimes. Such knowledge is crucial for developing targeted interventions and deriving appropriate recommendations for action.

## 2. Materials and Methods

### 2.1. Patient Cohort

The study was based on a cohort from the Lean European Open Survey on SARS-CoV-2-Infected Patients (LEOSS) [13]. The LEOSS project was established in March 2020 as a non-interventional, multicenter network focusing on data from hospitalized COVID-19 patients. A prerequisite for enrollment in the LEOSS registry was a confirmed diagnosis of COVID-19 disease (PCR or rapid antigen test as an acceptable alternative). More detailed information about LEOSS may be obtained from the project’s website (https://leoss.net, accessed on 8 March 2023) or the German Clinical Trials Register (DRKS, No. S00021145).

Anonymized patient data were retrospectively entered into the LEOSS registry upon termination of acute care, i.e., either when the treatment was finished or when the patient was deceased. The clinical data were reported by an electronic case report form (eCRF) utilizing an online platform, ClinicalSurveys.net, developed by the University Hospital Cologne (UHC), Germany, and hosted by QuestBack, Oslo, Norway, on servers at the UHC [14]. To guarantee anonymity throughout the entire analysis, a customized LEOSS scientific use file (SUF) was created based on the principles of the LEOSS public use file (PUF) described in Jakob et al. [14]. Both vertical (categorical scoring of numeric variables) and horizontal data aggregation (data aggregation within disease phases) were used to prevent re-identification. Four phases were used for categorization, which can be broadly characterized as asymptomatic/mild symptoms (uncomplicated phase), a need for oxygen supplementation (complicated phase), a need for critical care (critical phase), and the recovery phase. An in-depth description of the clinical phase definition as well as of the recorded data items are available on https://leoss.net (accessed on 8 March 2023) and in [15]. Patients of all ages were included. Age was recorded categorically. Age ranges were defined so that cases of adult patients could be examined in 10-year increments. For pediatric patients, smaller age increments were considered to reflect differences in developmental stages between age groups.

### 2.2. Study Design

The LEOSS case registry collects patient data from study sites in Austria, Belgium, Bosnia and Herzegovina, Germany, Italy, Latvia, Spain, Switzerland, Turkey, and the United Kingdom, with the vast majority of data coming from Germany. The present study focused on intensive care at the beginning of the COVID-19 pandemic, which was characterized by a health system overload (first wave of the pandemic until the transition to the second wave, i.e., March to October 2020 in Germany). To this end, all patients treated at either of the LEOSS partner centers between 23 March 2020 and 12 October 2020, who entered the critical phase as defined by the LEOSS database [15] at some point within onset of their COVID-19 disease, were fully enrolled, allowing a total number of 840 patients to be included. Critical phase was declared when at least one of the indicated criteria was met: the need for catecholamines, life-threatening cardiac arrhythmias, the need for unplanned mechanical ventilation (invasive or non-invasive), prolongation (>24 h) of planned mechanical ventilation, liver failure with Quick < 50% or INR > 3.5, a qSOFA score of ≥2, or acute renal failure with a need for dialysis. Interest was focused on a potential influence of age and comorbidity burden on the applied ventilation strategy and patient outcome. To this end, of the specific critical care data elements available from the LEOSS registry, the following data elements were analyzed: (i) patient characteristics (age, comorbidities, Charlson comorbidity index), (ii) the ventilation treatments performed (no ventilation, non-invasive ventilation, invasive ventilation, ECMO), and (iii) the outcome (recovery, in-hospital mortality).

### 2.3. Data Quality

To ensure the quality of the data, several plausibility checks were built into the eCRF during its construction, which generate warning messages in case of incorrect entries. In addition, medical staff from the LEOSS centers and the project group checked the accuracy and plausibility of the data both during entry and prior to data analysis.

There was no missing data regarding the following parameters analyzed: type of ventilation therapy, Charlson comorbidity index, and number of comorbidities. For the parameters age and outcome, the proportion of missing data was low (0.7% and 1.1%, respectively) and of the MCAR type (missing completely at random). In the statistical analysis, the missing was accepted and the corresponding cells were left blank.

### 2.4. Statistical Analysis

All data handling, the statistical analysis, and numerical calculations were performed with R (R Development Core Team, Vienna, Austria, version 4.1.1, 2021). Data were all reported as categorical variables (numbers and percentages). Survival was analyzed using Kaplan–Meier curves and log rank test. In addition, Cox regression was used to study the association between ventilation regime and survival, taking as reference the variable invasive ventilation with the largest size. Both univariate analysis and multivariable Cox regression were performed, adjusting for the potential confounders of age, number of comorbidities, and Charlson comorbidity index. Results were presented as hazard ratio (HR) with 95% confidence interval (CI). A log rank value *p* < 0.05 was considered for statistical significance.

## 3. Results

### 3.1. Characteristics of the Study Population

**Figure 1:**
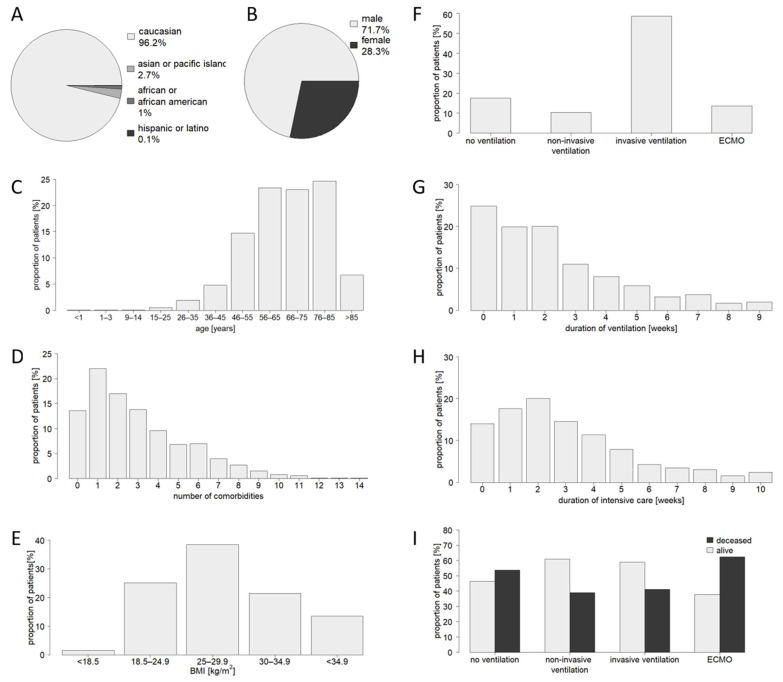
Characteristics of the study cohort (*n* = 840). (**A**): Ethnic distribution, (**B**): gender distribution, (**C**): age distribution, (**D**): distribution of comorbidity burden, (**E**): BMI distribution, (**F**): frequency of use of certain ventilation therapies, (**G**): duration of ventilation, (**H**): duration of intensive care, (**I**): hospital outcome by treatment group.

The study was based on aggregate SARS-CoV-2-positive patients admitted to an intensive care unit of a LEOSS study center during the study period (*n* = 840; Figure 1). The absolute majority of patients were Caucasian. There was also a clustering of patients of male gender and of patients older than 45 years of age. Median age was 66 to 75 years. The number of comorbidities documented for an individual patient ranged from 0 to 14, with only 13.9% of patients having no reported comorbidities and 22.0% of patients having only one reported comorbidity. More details of comorbidities are provided in Table 1. Normal weight was present in 25.2% of patients. In 73.4% of cases, BMI was elevated (>24.9), whereas underweight (BMI < 18.5) was seen in as few as 1.4% of cases. Median BMI was 25 to 29.9. Ventilation therapies performed included non-invasive ventilation (10.4%; 87/840; type of non-invasive ventilation not specified), invasive ventilation (58.5%; 492/840), and ECMO (13.6%; 114/840). A total of 147 patients (17.5%) did not receive any ventilation therapy. The documented duration of ventilation therapy ranged from 0 to 9 weeks. Treatment was performed in prone position in 8.0% of non-invasively ventilated patients, 62.0% of invasively ventilated patients, and 81.6% of ECMO-treated patients. In general, intensive care treatment was required for a period of 0 to 3 weeks in the majority of patients (66.2% of cases), but lengths of treatment of up to 10 weeks have also been recorded. The overall in-hospital mortality rate was 46.0%, with increased mortality specifically in the non-ventilated group (53.7%; 79/147) and the ECMO group (62.3%; 71/114).

### 3.2. Patient Age and Comorbidity Influence the Ventilation Strategy in Critical Care

Ventilation strategy is based on acute respiratory distress syndrome (ARDS) severity while considering clinical factors, such as organ dysfunction and frailty. The majority of intubated patients (73%) had moderate to severe ARDS (PaO2/FiO2 < 200 mmHg), while non-invasively ventilated patients had predominantly mild ARDS (PaO2/FiO2 200–300 mmHg). No non-invasively ventilated patients with severe ARDS were documented. Patients who were intubated also had more severe organ failure. Median sequential organ failure assessment (SOFA) scores were 12 for ECMO patients and 9.5 for invasively ventilated patients. A median SOFA score of three was documented in the non-ventilated and non-invasively ventilated groups. Data on frailty, as rated by the clinical frailty scale (CFS), were not available. Thus, patient age and comorbidity burden were used to assess the potential influence of patient factors on treatment decisions.

**Figure 2:**
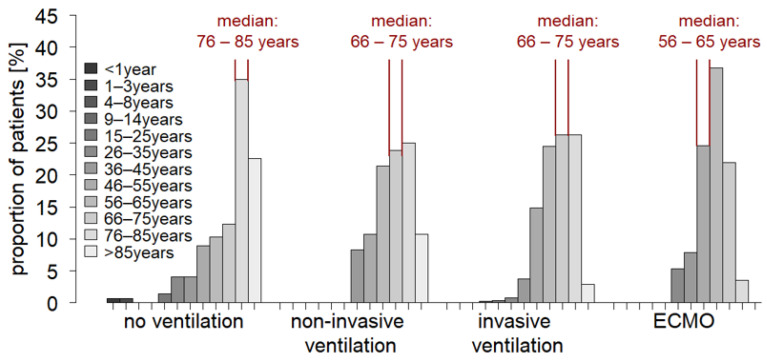
Distribution of age in COVID-19 patients in intensive care grouped by ventilation therapy received (total cohort, *n* = 840).

**Figure 3:**
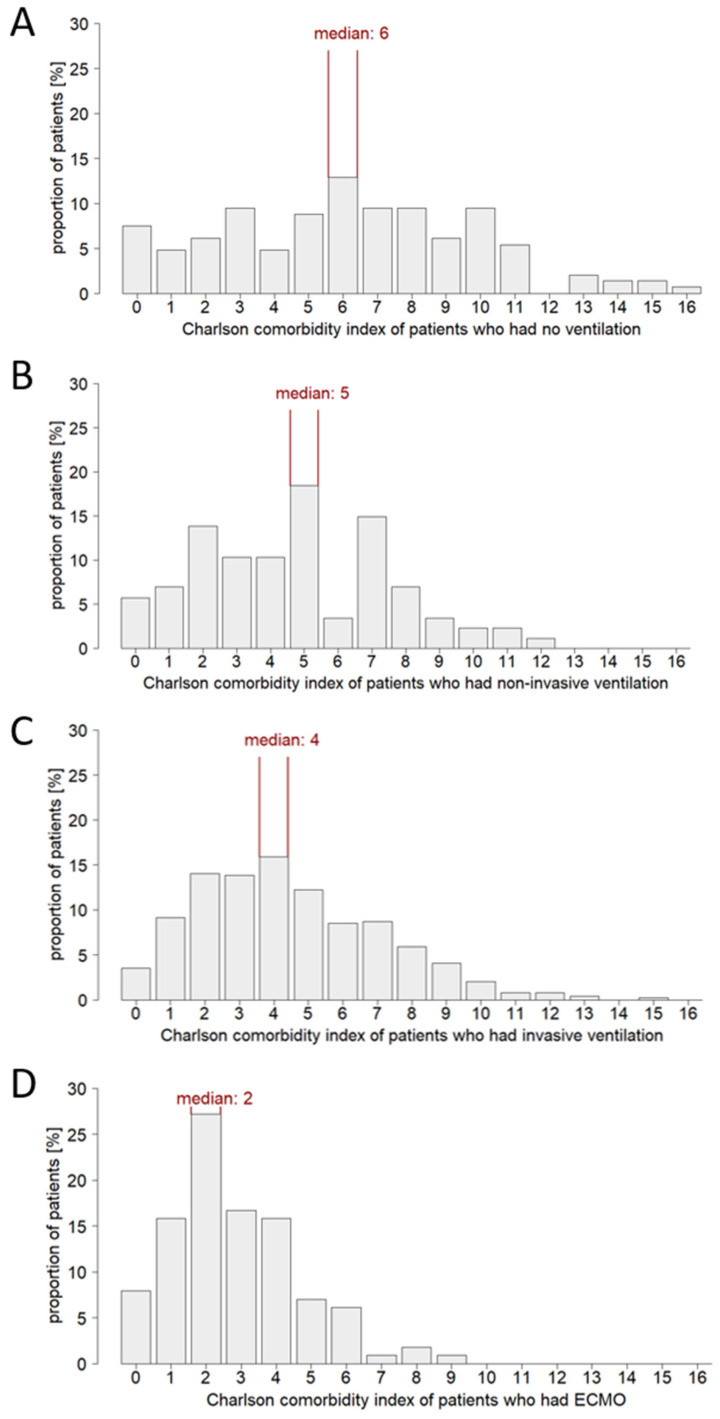
Distribution of the Charlson comorbidity index in COVID-19 patients receiving intensive care. (**A**): Sub-cohort of non-ventilated patients (*n* = 147), (**B**): sub-cohort of non-invasively ventilated patients (*n* = 87), (**C**): sub-cohort of invasively ventilated patients (*n* = 492), (**D**): sub-cohort of ECMO patients (*n* = 114).

**Figure 4:**
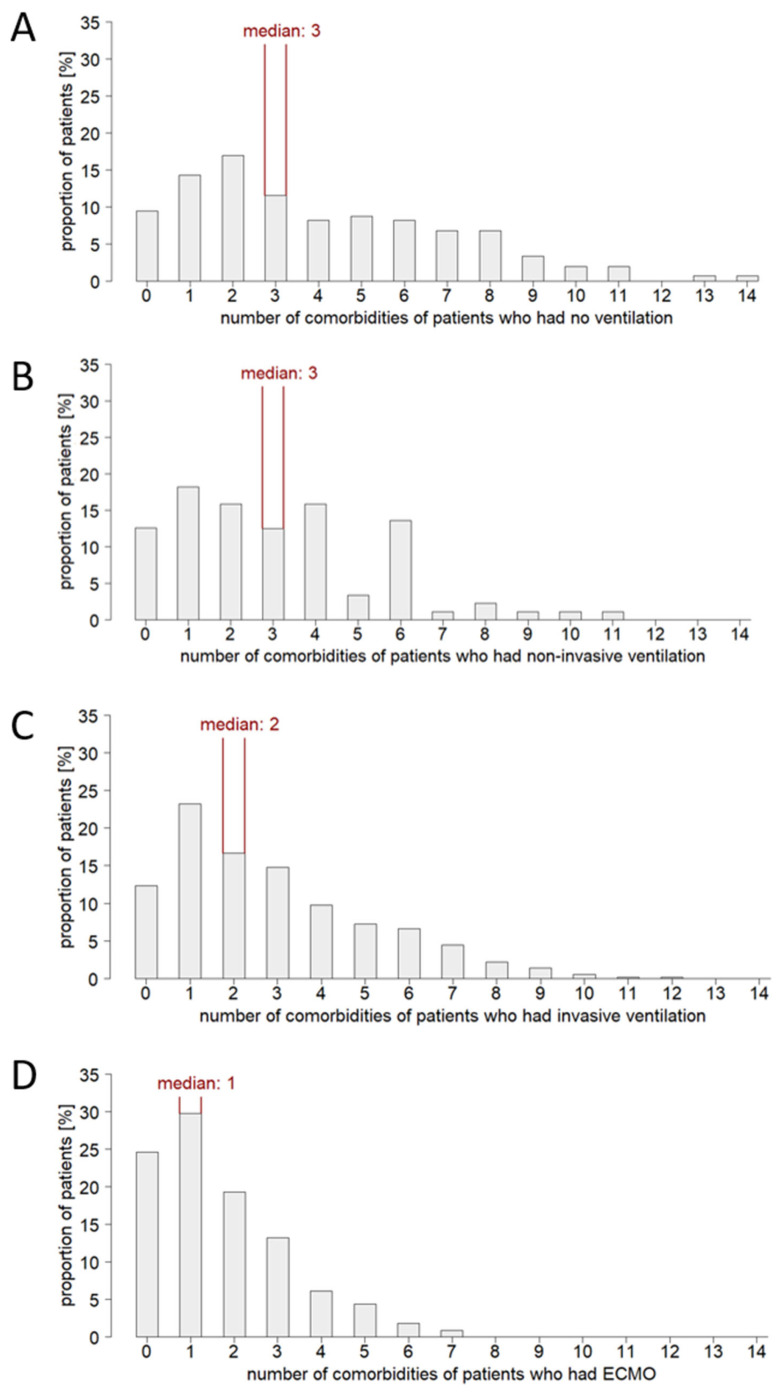
Distribution of the number of comorbidities in COVID-19 patients receiving intensive care. (**A**): Sub-cohort of non-ventilated patients (*n* = 147), (**B**): sub-cohort of non-invasively ventilated patients (*n* = 87), (**C**): sub-cohort of invasively ventilated patients (*n* = 492), (**D**): sub-cohort of ECMO patients (*n* = 114).

In the group of non-ventilated patients, there was a distinct rightward shift to higher age (Figure 2). In contrast, in the group of ECMO patients, a leftward shift to lower age was found and no patients of advanced age (>85 years) underwent ECMO procedure. Moreover, the majority of this patient group (58.9% of cases) was also not ventilated despite critical illnesses. It is also noted that in the few documented pediatric patients receiving intensive care, no ventilation was performed up to the age of 3 years. Substantial disparities were also observed with respect to Charlson comorbidity index (Figure 3). A widespread range of Charlson comorbidity index was found in the group of non-ventilated patients. However, in ventilated patients, there was a leftward shift to lower Charlson comorbidity index values with increasing invasiveness of therapy. Specifically, this was evident in the group of ECMO patients, indicating a cautious use of high-invasive ventilation techniques in a setting of severe morbidity burden. Actually, the group of ECMO patients was characterized by a below-average comorbidity burden (Figure 4). No comorbidities were found in 24.6% of cases and only one comorbidity in 29.8% of cases. The maximum number of comorbidities reported for individual ECMO patients was seven (compared with fourteen in the non-ventilated group, eleven in the non-invasively ventilated group, and twelve in the invasively ventilated group). Although this seems contradictory at first, this observation might relate to the age structure of this patient cohort. Patients of advanced age (>85 years), typically characterized by a high comorbidity burden, were primarily treated non-invasively and did not receive ECMO therapy in any case. Overall, the data suggested a preselection in treatment decisions. Unfortunately, the LEOSS dataset does not include information on advance directives. Thus, it is not possible to assess the extent to which the observed differences are due to a possible higher proportion of patient-desired limitation of life-sustaining measures (LLST) in elderly, multimorbid patients.

### 3.3. Patient Age and Comorbidity Have an Impact on the Outcome of Critical Care Treatment

**Figure 5:**
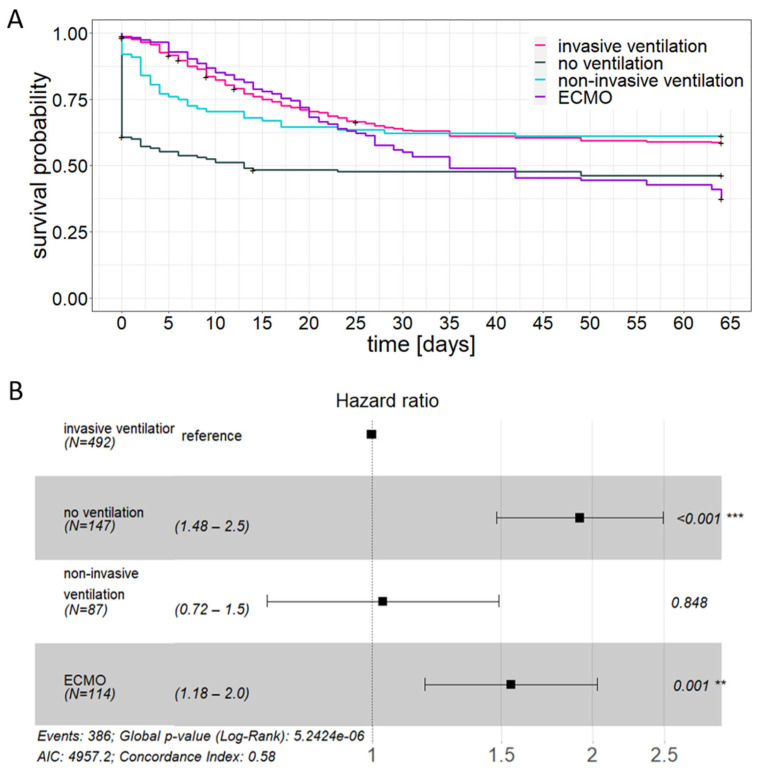
Association between ventilation regime and survival. (**A**): Unadjusted Kaplan–Meier analysis, (**B**): Forest plot depicting univariate Cox regression. ** *p* < 0.01, *** *p* < 0.001.

In total, 386 of 840 patients (46.0%) died during their hospitalization with differences between ventilation groups: death was significantly more common in non-ventilated patients and ECMO-treated patients compared to patients receiving non-invasive or invasive ventilation (Figure 5A). Indeed, univariate analysis showed an effect of ventilation regimen on mortality (Figure 5B). This is also reflected in the documented 30-day mortality and median survival time for the treatment groups (Table 2). While the majority of both non-invasively and invasively ventilated patients reached the recovery phase, the median survival time of ECMO-treated patients was 35 days and that of non-ventilated patients was only 13 days.

**Figure 6:**
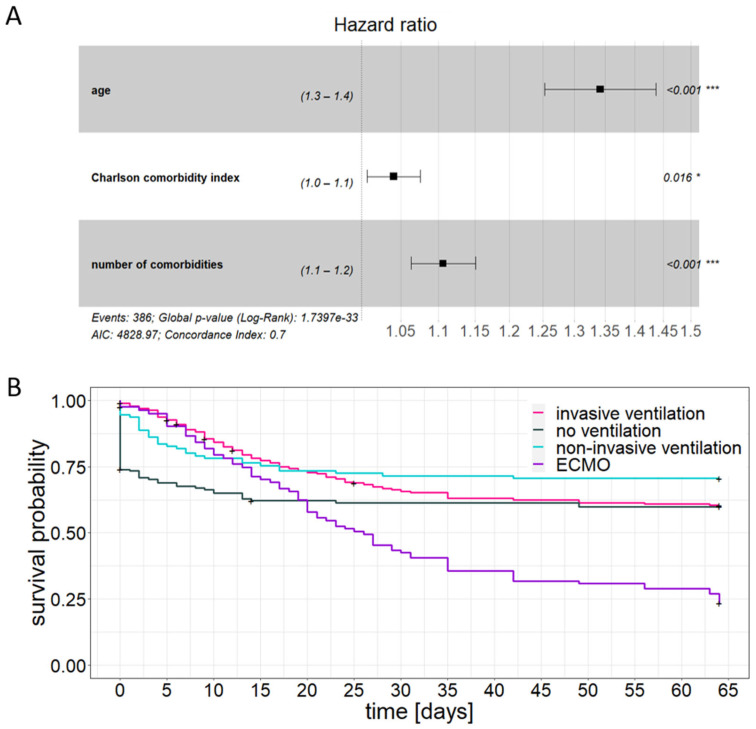
Confounding factors age, Charlson comorbidity index, and number of comorbidities. (**A**): Forest plot depicting multivariable Cox regression, (**B**): adjusted Kaplan–Meier analysis. * *p* < 0.05, *** *p* < 0.001.

Multivariable adjustment for clinical variables demonstrated that, in addition to the well-known confounders “ARDS severity” (HR horovitz index: 1.279; 95% CI 1.034–1.582) and “organ dysfunction” (HR SOFA score: 1.072; 95% CI 1.006–1.142), age and comorbidity burden also have an influence (Figure 6A). Accordingly, Kaplan–Meier survival analysis adjusted by the confounding factors age, Charlson comorbidity index, and number of comorbidities, revealed distinct alterations regarding 30-day mortality and median survival time, which specifically concerned the non-ventilated and the ECMO-treated patients (Figure 6B, Table 2). A median survival time could only be determined for the ECMO-treated group and was reduced to 26 days. In contrast, the adjusted 30-day mortality of the non-ventilated group approached that of the invasively ventilated group.

**Figure A1:**
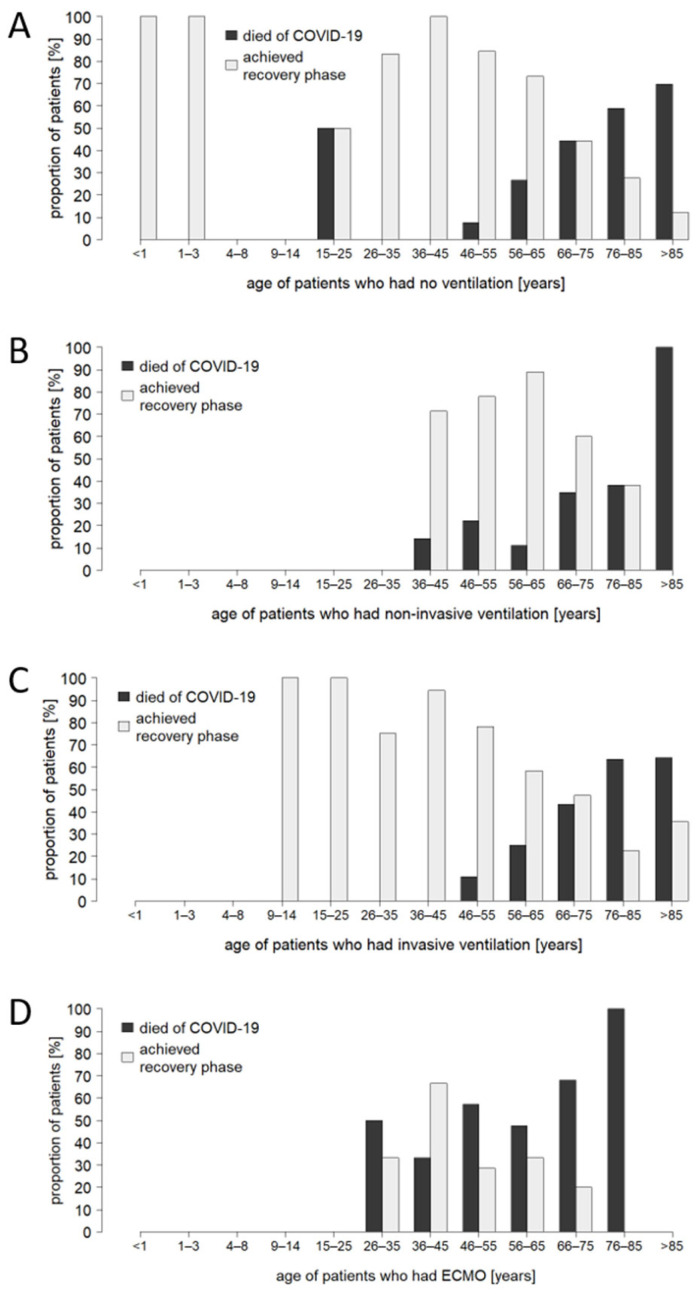
Distribution of age in recovered and deceased COVID-19 patients receiving intensive care. (**A**): Sub-cohort of non-ventilated patients (*n* = 147), (**B**): sub-cohort of non-invasively ventilated patients (*n* = 87), (**C**): sub-cohort of invasively ventilated patients (*n* = 492), (**D**): sub-cohort of ECMO patients (*n* = 114).

The impact of age on outcome is clearly seen when comparing the age distribution of deceased and recovered patients. The risk of death in hospital increases with age regardless whether patients were ventilated non-invasively, invasively, or additionally treated by ECMO. However, depending on the invasiveness of the therapy, there was a shift in the age at which the turning point in the ratio between recovered and deceased patients was reached (Figure A1). While in the group of non-invasively ventilated patients, a majority of deaths were documented only from the age >85 years, whereas in the group of invasively ventilated patients, this was already the case from the age group 76–85 years, and in ECMO patients from the age group 46–55 years.

**Figure A2:**
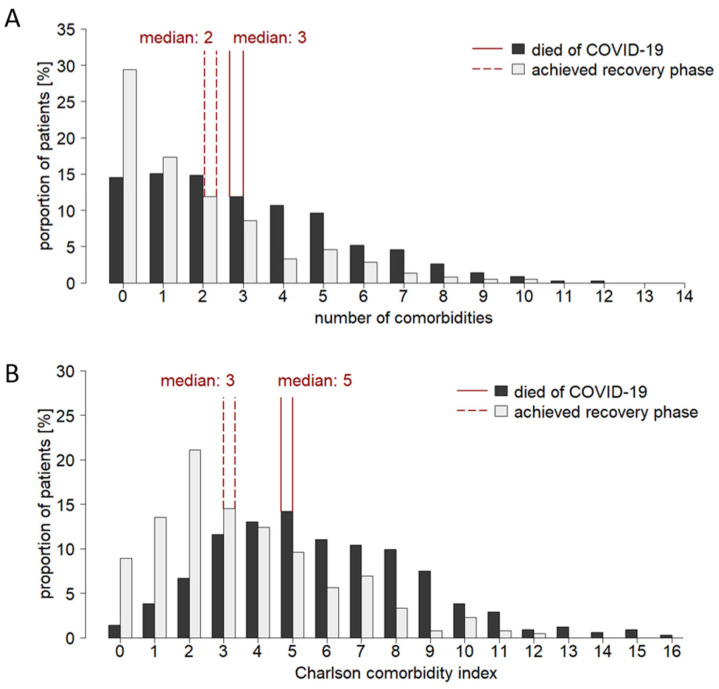
Distribution of (**A**) the number of comorbidities and (**B**) the Charlson comorbidity index in recovered and deceased COVID-19 patients receiving intensive care (total cohort, *n* = 840).

The comparison of deceased and recovered patients also reveals the influence of comorbidity burden on outcome. For COVID-19 patients with no or only one documented comorbidities, the proportion who reached the recovery phase was higher than the proportion who died. However, starting with a documented number of two comorbidities, this ratio reversed (Figure A2A). Likewise, a rightward shift of the Charlson comorbidity index to higher values was observed in deceased patients compared to recovered patients (Figure A2B). In the recovered group, values ranging from 0 to 12 were documented, with the majority of patients (21.1%) having a Charlson comorbidity index of two. This contrasts with the group of deceased patients, where a Charlson comorbidity index of two was documented in only 6.7%, and values as high as sixteen were reported. The association between a high number of comorbidities or a high Charlson comorbidity index and an increased mortality risk was evident for all sub-cohorts by ventilation type. Certain comorbidities clustered in patients who died of COVID-19. These were primarily cardiovascular comorbidities (chronic heart failure, atrial fibrillation, coronary artery disease, aortic stenosis, and hypertension). However, pulmonary comorbidities (chronic lung disease) and metastatic solid tumors were also significantly more common in deceased patients.

## 4. Discussion

The present study addressed the impact of the inherent factors of critically ill patients with COVID-19, namely age and comorbidity burden, on the ventilation therapy applied on ICU as well as treatment outcome. The resulting data underscore the relevance of both confounding factors. Remarkably, the influence was twofold. First, highly advanced age and multimorbidity were associated with the restrictive use of invasive ventilation therapies, specifically ECMO. This may have contributed to the relatively high mortality observed in the sub-cohort of non-ventilated ICU patients. On the other hand, as invasiveness of ventilation therapy increased, the age at which treatment was successfully completed by the majority of patients declined.

The S3 guideline “Recommendations for inpatient therapy of patients with COVID-19” (AWMF registry number 113/001; [16]), which applies in Germany and therefore to the majority of patients in the LEOSS registry, recommends an apparatus-based therapy escalation in acute respiratory failure due to COVID-19. In case of progressive deterioration of gas exchange and increased oxygen demand (PaO2/FiO2 < 150 mmHg and respiratory rate >30/min) accompanied by organ dysfunction, intubation and invasive ventilation should be considered. The implementation of these recommendations is reflected in the study cohort. The majority of patients with severe ARDS and a high degree of organ dysfunction were intubated. However, the data also suggest that not only disease severity, but also age and comorbidity burden may have contributed to the treatment choice. ECMO was limited for patients older than 3 years and younger than 85 years. In contrast, a clustering of individuals older than 76 years was observed in the sub-cohort of non-ventilated patients. A shift was also seen in terms of patient comorbidity burden: the more invasive a ventilation option, the lower the comorbidity burden of the patients receiving it. Thus, in the sub-cohort of non-ventilated patients, individuals with a Charlson comorbidity index up to 16 and a total number of documented comorbidities up to 14 were found. In the sub-cohort of ECMO patients, however, the maximum Charlson comorbidity index was nine and the maximum comorbidity count was seven. Apparently, geriatric, multimorbid patients were treated less aggressively without exhausting all treatment options. One can assume that the limited ICU capacities in the first acute COVID-19 pandemic wave added to the reserved usage of invasive ventilation. On the other hand, it is known that about 50% of the elderly population (>60 years) in Germany has composed an advance directive [17]. Therefore, it can be speculated that the limited use of mechanical ventilation in this patient group was primarily in response to patient wishes rather than based on a physician triage system. In fact, a recent study of elderly (≥80 years) ICU patients reported more frequent withholding or withdrawal of life-sustaining measures in COVID-19 patients compared to non-COVID-19 patients [18]. The same study also found an increased 30-day mortality in COVID-19 patients compared to non-COVID-19 patients. However, it remains unclear whether this finding reflects a more active policy of withholding treatment or an inherent increased mortality risk due to COVID-19 [18]. Overall, these findings highlight the need for comprehensive research on LLST. Critical care databases should include advance directives as a mandatory data point. Healthcare professionals’ assessment of a patient’s risk-benefit profile may be another factor which is worthy of discussion. In the S3 guideline referred to above, it is stated that clinical factors, including age and comorbidities, should be considered when deciding whether to intubate a patient [16]. In addition to the assessment of severity, frailty is often used as a decision-making aid [2,19,20]. This approach raises the question of whether age and multimorbidity are not only risk factors for needing intensive care [1,2,3,4,5,6,8,21,22,23,24], but also influence the success of certain intensive care interventions.

The present study clearly demonstrates that age and comorbidity burden affect the outcome of intensive care treatment of COVID-19 patients. Remarkably, the age at which treatment could be completed with survival in the majority of patients was observed to shift in relation to the invasiveness of the ventilation therapy performed: the more invasive a ventilation option, the earlier the turning point was reached. Apparently, age, therapeutic intervention, and treatment success were interlinked. A critical factor for treatment success is a patient’s disease severity. Mechanical ventilation and, even more so, ECMO are used in patients with a serious course of disease, which per se implies an elevated mortality risk. Multiple mechanisms discussed to contribute to more severe disease progression are age-associated. These include pre-existing malfunctions, immune senescence, age-related limitations of lung function, the coagulation system, and the endothelial barrier, as well as imbalances in nutritional status and intestinal dysbiosis, which are more common in the elderly [4,5,7,8]. Another influencing factor is the violence of the therapy performed. With the increasing invasiveness of a treatment, the probability of undesirable side effects rises, which may negatively affect the outcome. It is known that advanced age elevates the risk of such side effects [10]. Therefore, the harm–benefit balance of invasive ventilation strategies becomes rather critical with age.

The group of non-ventilated patients was characterized by a high proportion of very old, multimorbid individuals and also by a substantial mortality rate. The available data do not allow us to conclusively determine whether a more aggressive treatment of these patients would have been associated with better outcomes. Nonetheless, the findings of this study underscore the importance of a holistic approach in decision-making to ensure that treatment is proportionate and meets the patient’s wishes. Advanced age is a relative (not an absolute) contraindication to the use of ECMO, although no threshold has been established [25]. In general, the decision to use invasive ventilation therapies, such as mechanical ventilation or ECMO, should be made after careful consideration of potential benefits and harms, especially in patients of advanced age [25,26]. Chronological age is not a good indicator of outcome here. Rather, the patient’s health status should also be considered. Accordingly, the frailty of a patient is discussed as a suitable prognostic marker [2,4,5,6,7,9,11,27]. Specifically, the use of the clinical frailty scale (CFS) is recommended for priority setting, decision-making, and pandemic triage [2,4,6,9,11]. As a caveat, focusing on CFS (in analogy to the traditional focus on patient age) has the potential to perpetuate established patterns of inequity. This is especially true for older, frail individuals who desire comprehensive intensive care. The extent to which CFS played a role in decision-making in the study population cannot be estimated because CFS data were not available from the patients.

Our analyses based on LEOSS have the advantage of a standardized protocol and data from different regions and sectors. However, the majority of patients included were from Germany, limiting the generalizability of our results. A clear limitation of our study is its retrospective observational nature. The LEOSS registry did not collect data on patients’ frailty as assessed by CFS. The lack of knowledge about whether patients were frail and to what extent severely limits the interpretation of the data. Information on the type of non-invasive ventilation used was also not available. It was not possible to determine the prevalence of advance directives among ICU patients because this element was included in LEOSS at a later stage. Our data represent patients recruited during the first pandemic wave. The extent to which the data are different from patients who were in intensive care for SARS-CoV-2 infection later in the pandemic is unknown but may be of interest for investigation.

## 5. Conclusions

Our study highlights the impact of age and comorbidity burden on the outcome of COVID-19 patients receiving intensive care. Our data further point toward a relationship between the type and invasiveness of a therapeutic measure, the patient age, and the outcome. The more aggressive an applied procedure, the younger the age in which a majority of patients died in hospital. In addition, our study spotlights that specifically geriatric and multimorbid patients are predominately excluded from invasive ventilation regimens, such as ECMO, thus precluding an assessment of the potential benefit of these therapeutic approaches for that patient population.

## Figures and Tables

**Table 1:** Comorbidities of the study cohort (*n* = 840).

Comorbidity	No. (%)
Hypertension	512 (61.0)
Diabetes without end-organ damage	155 (18.5)
Chronic kidney disease	145 (17.3)
Coronary artery disease	140 (16.7)
Atrial fibrillation	134 (16.0)
Chronic heart failure	94 (11.2)
Chronic obstructive pulmonary disease (COPD)	83 (9.9)
Diabetes with end-organ damage	81 (9.6)
Acute kidney injury	80 (9.5)
Cerebrovascular disease	78 (9.3)
Solid tumor	73 (8.7)
Myocardial infarction	64 (7.6)
Dementia	63 (7.5)
Chronic pulmonary disease	51 (6.1)
Peripheral vascular disease	42 (5.0)
On dialysis	34 (4.0)
Asthma	31 (3.7)
Carotid artery disease	31 (3.7)
Rheumatic disease	30 (3.6)
Hemiplegia	27 (3.2)
Lymphoma	27 (3.2)
Atrioventricular block	25 (3.0)
Chronic liver disease	25 (3.0)
Organ transplantation	20 (2.4)
Peptic ulcer	20 (2.4)
Aortic stenosis	18 (2.1)
Leukemia	15 (1.8)
Solid tumor, metastasized	12 (1.4)
Liver cirrhosis	7 (0.8)

**Table 2:** Median survival time and 30-day mortality by ventilation therapy received before and after adjustment for the confounding factors age, Charlson comorbidity index, and number of comorbidities.

	Median Survival Time [Days]		30-Day Mortality [%]	
	unadjusted	adjusted	unadjusted	adjusted
no ventilation	13	-	52.4	38.6
non-invasive ventilation	-	-	37.7	28.4
invasive ventilation	-	-	36.6	34.5
ECMO	35	26	44.9	57.5

## Data Availability

Patient data from the LEOSS registry are subject to the LEOSS governance, data use, and access policy (policy text available on https://leoss.net, accessed on 8 March 2023).
